# Cannflavins A and B with Anti-Ferroptosis, Anti-Glycation, and Antioxidant Activities Protect Human Keratinocytes in a Cell Death Model with Erastin and Reactive Carbonyl Species

**DOI:** 10.3390/nu15214565

**Published:** 2023-10-27

**Authors:** Huifang Li, Ni Deng, Tess Puopolo, Xian Jiang, Navindra P. Seeram, Chang Liu, Hang Ma

**Affiliations:** 1 Bioactive Botanical Research Laboratory, Biomedical and Pharmaceutical Sciences, College of Pharmacy, University of Rhode Island, Kingston, RI 02881, USA; 2 Department of Dermatology, Laboratory of Dermatology, Clinical Institute of Inflammation and Immunology, Frontiers Science Center for Disease-Related Molecular Network, West China Hospital, Sichuan University, Chengdu 610041, China; 3 Proteomics Facility, College of Pharmacy, University of Rhode Island, Kingston, RI 02881, USA

**Keywords:** skin-aging, ferroptosis, reactive carbonyl species, proteomics, cell death, keratinocytes

## Abstract

Precursors of advanced glycation endproducts, namely, reactive carbonyl species (RCSs), are aging biomarkers that contribute to cell death. However, the impact of RCSs on ferroptosis—an iron-dependent form of cell death—in skin cells remains unknown. Herein, we constructed a cellular model (with human keratinocyte; HaCaT cells) to evaluate the cytotoxicity of the combinations of RCSs (including glyoxal; GO and methyglyoxal; MGO) and erastin (a ferroptosis inducer) using bioassays (measuring cellular lipid peroxidation and iron content) and proteomics with sequential window acquisition of all theoretical mass spectra. Additionally, a data-independent acquisition approach was used to characterize RCSs’ and erastin’s molecular network including genes, canonical pathways, and upstream regulators. Using this model, we evaluated the cytoprotective effects of two dietary flavonoids including cannflavins A and B against RCSs and erastin-induced cytotoxicity in HaCaT cells. Cannflavins A and B (at 0.625 to 20 µM) inhibited ferroptosis by restoring the cell viability (by 56.6–78.6% and 63.8–81.1%) and suppressing cellular lipid peroxidation (by 42.3–70.2% and 28.8–63.6%), respectively. They also alleviated GO + erastin- or MGO + erastin-induced cytotoxicity by 62.2–67.6% and 56.1–69.3%, and 35.6–54.5% and 33.8–62.0%, respectively. Mechanistic studies supported that the cytoprotective effects of cannflavins A and B are associated with their antioxidant activities including free radical scavenging capacity and an inhibitory effect on glycation. This is the first study showing that cannflavins A and B protect human keratinocytes from RCSs + erastin-induced cytotoxicity, which supports their potential applications as dietary interventions for aging-related skin conditions.

## 1. Introduction

Carbonyls are highly reactive electrophilic compounds that can be endogenously generated from the metabolism of carbohydrates, lipids, and amino acids. Biologically derived carbonyls, such as dicarbonyl species including glyoxal (GO) and methylglyoxal (MGO), are metabolites from the oxidation of carbohydrates (i.e., glycolysis). Due to their electrophile moieties (α,β-unsaturated aldehyde and ketone), these reactive carbonyl species (RCS) can readily undergo nonenzymatic reactions with proteins and lipids to form a group of detrimental molecular complexes known as advanced glycation endproducts (AGEs). Dicarbonyl species are also prone to react with DNA to form GO/MGO-DNA adducts, which can result in DNA damage [1]. In the cellular environment, GO and MGO can attack cell membranes and disrupt the biological functions of essential cell components including membrane transporters, enzymes, and signaling pathways [2,3]. In addition, an abnormal influx of GO and MGO contributes to spiked cellular oxidative stress, which can further lead to several cell dysfunctions including various forms of cell death. For instance, MGO was reported to disturb redox-homeostasis in human keratinocytes by inducing mitochondrial oxidative stress and leading to apoptosis-mediated skin cell death [4]. Apart from inducing cell death, MGO can also function as a sensitizer to exacerbate ultraviolet-induced oxidative stress and apoptosis in keratinocytes [5]. Moreover, high levels of GO- and MGO-derived AGEs, namely, GOLD (GO-lysine dimer) and MOLD (MGO-lysine dimer), are biomarkers of undermined skin barrier function and accelerated skin aging [6,7].

Notably, skin aging is also associated with a more recently (in 2012) discovered form of cell death, namely, ferroptosis [8]. Ferroptosis, also known as oxytosis, is an iron-dependent cell death characterized by accumulated lipids peroxidation. It is regulated by a cysteine (an endogenous antioxidant)-mediated cellular redox system, which is responsible for the biosynthesis of reduced glutathione (GSH) [9]. GSH serves as a cofactor for several antioxidant enzymes including glutathione peroxidase 4 (GPX4), which converts lipid peroxides into non-toxic lipid alcohols. The biosynthesis of GSH is dependent on cysteine transported by a cystine–glutamate antiporter (i.e., system Xc-). Thus, the inhibition of system Xc- by small molecules, for instance, a synthetic compound named erastin, can disrupt the production of endogenous antioxidants and impair cellular redox-homeostasis, eventually leading to ferroptosis [10]. Although it has been reported that MGO detoxification-related proteins, such as protein deglycase DJ-1, are associated with the inhibition of erastin-triggered ferroptosis in DJ-1−/− mouse embryonic fibroblast cells [11], the role of dicarbonyls in ferroptosis in keratinocytes remains unclear.

Published studies support that plant-based dietary polyphenols are potential interventions of aging-related skin disorders [12,13,14]. Several mechanisms, such as antioxidant [15], anti-glycation [16], RCS-trapping [17], DNA protection [18], and the inhibition of RAGE-mediated inflammation [19], are involved in dietary polyphenols’ anti-skin-aging effects. Our laboratory has conducted systematic evaluations of polyphenols for skin protective effects. We have reported that dietary polyphenols including gallotannins from red maple (Acer rubrum) and ellagitannins pomegranate (Punica granatum) can inhibit the formation of AGEs [20,21] and reduce oxidative stress-induced cytotoxicity in human keratinocytes (HaCaT cells) [4,22,23,24]. The combination of maple gallotannin and pomegranate ellagitannin exerted protective effects on human skin collagen against AGEs-induced cross-linking [25]. In addition, we reported that a phytochemical from cannabis, namely, cannabidiol, showed antioxidant and cytoprotective effects against ferroptosis in HaCaT cells. However, it is not clear whether other phytochemicals from cannabis, such as cannflavins, a group of unique cannabis flavonoids, can protect skin cells from oxidative stress-induced cell death.

**Scheme 1:**
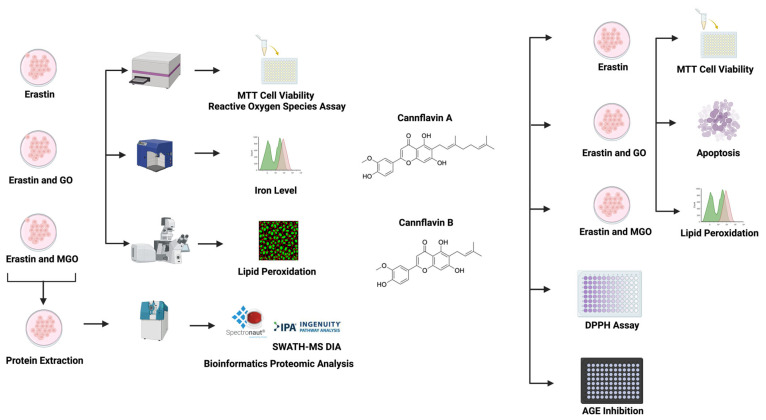
Workflow for the cellular bioassays to evaluate the effects of GO and MGO on erastin-induced ferroptosis in human keratinocytes by examining ferroptosis-related biomarkers including cell viability, ROS production, cellular iron levels, and lipid peroxidation. Next, a SWATH-MS-based DIA was conducted for proteomic analyses to identify molecular targets (including proteins and pathways) that were involved in ferroptosis with or without the presence of GO or MGO in HaCaT cells exposed to erastin, GO, MGO, and their combinations. Lastly, the cytoprotective effects of CFA and CFB against erastin and combinations of erastin + GO- and erastin + MGO-induced cell death. This scheme is created with BioRender (https://app.biorender.com/; accessed on 16 July 2023; Toronto, Ontario, Canada).

Herein, we aimed to use cellular and proteomics-based assays to delineate the overall impact of GO and MGO on erastin-induced ferroptosis in human keratinocytes. The biological effects of GO and MGO on the cell viability of erastin-exposed keratinocytes were assessed, followed by the measurement of characteristic ferroptosis biomarkers including cellular lipid peroxidation and iron levels. In addition, a sequential window acquisition of all theoretical (SWATH) mass spectra (MS) data-independent acquisition (DIA), which is an advanced technique for the studies of biological processes, protein biomarker discovery, and drug target identification [26] was applied for proteomic analyses. We conducted SWATH-based proteomics to identify proteins and pathways that were associated with skin pathological conditions by comparing the proteomes of keratinocytes insulted with a ferroptosis inducer (erastin) in the presence or absence of GO and MGO. Furthermore, using the cell death model with erastin and RCSs, we evaluated the cytoprotective effects of cannflavins A and B (CFA and CFB, respectively) against erastin and combinations of erastin + GO- and erastin + MGO-induced cell death (Scheme 1).

## 2. Materials and Methods

### 2.1. Chemicals

Reagents including 3-(4,5-dimethylthiazol-2-yl)-2,5-diphenyltetrazolium bromide (MTT), dimethyl sulfoxide (DMSO), phosphate-buffered saline (PBS), glyoxal (GO), methylglyoxal (MGO), human serum albumin (HSA), 2′,7′-dichlorofluorescin diacetate (DC-FDA), and 2,2-diphenyl-1-picrylhydrazyl (DPPH) were purchased from Sigma-Aldrich (St. Louis, MO, USA). Cannflavin A (CFA) was prepared from hemp extracts using a method we previously reported [27]. The CFA used in this study was isolated from an extract of the leaves and flowers parts of mature hemp (*C. sativa*), from a hemp farm located in Kunming (Yunnan Province, China). Cannflavin B (CFB) was purchased from Emerald Scientific (San Luis Obispo, CA, USA). Liperfluo and FerroOrange were purchased from Dojindo Molecular Technologies (Rockville, MD, USA).

### 2.2. Cell Culture and Viability

Human keratinocytes (HaCaT cells) were procured from American Type Culture Collection (Rockville, MD, USA) and cultured as per the manufacturer’s instructions. The cell viability of HaCaT cells was evaluated using the MTT assay with minor modifications [25,28]. HaCaT cells were seeded in 96-well plates at a density of 1 × 10^4^ cells per well and allowed to adhere overnight. Next, cells were treated with erastin (20 µM) in the presence or absence of MGO (100 µM) or GO (100 µM), and then incubated for 24 h, followed by adding MTT solution to each well and incubated for 4 h. Then, the medium was removed and DMSO (100 µL) was added to dissolve the formazan salt crystals, followed by measuring the absorbance of each well at 570 nm using a SpectraMax M2 plate reader (Molecular Devices; Sunnyvale, CA, USA).

### 2.3. Measurement of ROS Level

The ROS level of HaCaT cells was evaluated using the staining reagents as we previously reported in an assay with minor modifications [29]. HaCaT cells were seeded in 96-well plates at a density of 1 × 10^4^ cells per well and allowed to adhere overnight. Next, cells were treated with CFA or CFB for 4 h, erastin (20 µM) in the presence or absence of MGO (100 µM) or GO (100 µM), and then incubated for 24 h. The medium was then removed, and the cells were incubated with fluorescent agent, DCF-DA (20 μM), for 30 min. The cellular fluorescence intensity was measured with an excitation and emission wavelength of 485 and 525 nm, respectively, using a SpectraMax M2 plate reader.

### 2.4. Cellular Lipid Peroxide Assessment

HaCaT cells were seeded in 6-well plates at a density of 3 × 10^5^ cells per well and allowed to attach overnight. The medium was then removed, and cells were treated with CFA and CFB for 4 h, then erastin in the presence or absence of MGO or GO for 24 h. Next, serum-free culture medium (200 μL) containing Liperfluo (5 μM) was added to each well, and then cells were incubated at 37 °C for 30 min. Cells were then washed with PBS and collected for flow cytometric analyses [30].

### 2.5. Detection of the Cellular Iron Level

HaCaT cells were seeded in 6-well plates at 3 × 10^5^ cells per well and allowed to attach overnight. Then, the medium was removed, and cells were incubated with CFA and CFB for 4 h, then erastin in the presence or absence of MGO or GO for 4 h. Next, cells were washed with serum-free culture medium, and then incubated with FerroOrange (3 μM) at 37 °C for 30 min. Then, the iron level was detected by a flow cytometric assay [31].

### 2.6. Homogenate Preparation

HaCaT cells were seeded in 100 mm dishes at 4.5 × 10^6^ per dish and allowed to attach overnight. Then, the medium was removed, and cells were incubated with erastin in the presence or absence of MGO and GO for 24 h. The medium was removed, and cells were washed with PBS twice, then collected in PBS after centrifuging at 1000× *g* for 10 min at 4 °C. The cell pellets were obtained and resuspended in homogenization buffer (150 μL; 8 M urea, 50 mM triethylammonium bicarbonate), and homogenized for 15 s. Cellular proteins were then centrifuged at 14,000× *g* for 10 min at 4 °C and the supernatant was collected. The total protein concentration was determined using a Pierce BCA protein assay kit (ThermoFisher Scientific; Waltham, MA, USA).

### 2.7. Trypsin Digestion

Protein digestion was conducted as described previously with modifications [32]. In this study, cell homogenates (n = 4, about 250 μg protein per sample) were denatured with 25 μL DTT (100 mM) at 34 °C for 30 min in a shaking water bath (1 rcf). Samples were alkylated by adding 25 μL IAA (iodoacetamide, 200 mM) in the dark at room temperature for 30 min. Then, samples were concentrated using the cold water, methanol, and chloroform (1:2:1, *v*/*v*/*v*) precipitation method followed by centrifugation at 10,000 rpm, 5 min at 10 °C. All the solvent was removed and ice-cold methanol (200 μL) was added to wash the protein pellet. The pellet was then suspended in ammonium bicarbonate (130 μL; 50 mM; pH = 8) containing 3% (*w*/*v*) sodium deoxycholate (DOC). Next, samples were spiked with TPCK-treated trypsin (10 μg) and transferred into barocycler tubes (PCT MicroTubes, Pressure Biosciences Inc.; Easton, MA, USA). The barocycler was run at 35 °C for 75 cycles with a 60 s pressure cycle (50 s high pressure, 10 s ambient pressure, and 25 kpsi). Following the first run, 10 μg trypsin was added to each sample, and the barocycler was run again at the above settings. After the barocycler, 15 μL of 5% formic acid (1:1 LC-MS grade acetonitrile and water; *v*/*v*) was added to 135 μL of digested peptide samples to precipitate DOC. The supernatant was collected after samples were centrifuged at 10,000 rpm for 5 min at 10 °C. Subsequently, 25 μL of the digested peptide sample was injected for LC-MS/MS analysis.

### 2.8. LC-MS/MS with SWATH Acquisition

Data were acquired on an AB Sciex TripleTOF 5600 mass spectrometer using a DuoSpray™ ion source (AB Sciex, Framingham, MA, USA) coupled to an Acquity H Class HPLC system (Waters Corp., Milford, MA, USA). An Acquity UPLC Peptide BEH C18 column (2.1 × 150 mm^2^, 300 Å, particle size 1.7 μm) with a VanGuard pre-column (2.1 × 5 mm^2^, 300 Å, particle size 1.7 μm) was used for the separation. The column temperature was set to 50 °C and the autosampler was set to 10 °C. Sample separation was employed within a 180 min gradient solvent method with a flow rate of 10 μL/min. Mobile phase A was water containing 0.1% (*w*/*v*) formic acid and mobile phase B was acetonitrile containing 0.1% (*w*/*v*) formic acid. A solvent composition scheme was used as follows: 98% A, 0–5 min; 98% to 70% A, 5–155 min; 70% to 50% A, 155–160 min; 50% to 5% A, 160–170 min; and 5% to 98% A held from 170–175 min. The gradient was returned to initial conditions from 170 to 175 min to equilibrate the column between samples. β-galactosidase (SCIEX, Framingham, MA, USA) was injected in every four samples for mass calibration. Data were acquired using the software Analyst TF (1.7.1). The DIA-based SWATH-MS was used for data acquisition. A positive ion mode was used for protein determination. The ESI source parameters were used as follows: ion source gas 1: 55; ion source gas 2: 60; curtain gas: 25 psi; ion spray voltage floating: 5500 V; The source temperature: 450 °C; Declustering potential: 100; collision energy: 10; and collision energy spread: 15; SWATH data were acquired within a mass range of *m*/*z* 400–1100 over 70 SWATH windows per cycle with a window size of *m*/*z* 10.

### 2.9. Data Processing and Data-Independent Acquisition Analysis

The raw WIFF files were converted to HTRMS files using the HTRMS converter and imported into Spectronaut version 16 (Biognosys, Schlieren, Switzerland). A human FASTA file downloaded from UniProt was used to generate the data library. The experiment consisted of 16 samples divided into four groups, each with four biological replicates. The Spectronaut settings were kept at their default values. Trypsin-P was selected as the protease specificity, while carbamidomethyl was set as the fixed modification. Acetyl (protein n-term) and oxidation were considered variable modifications. The peptide, protein, and peptide-spectrum match levels were set to a false discovery rate of 0.01. The XIC RT window was set to dynamic, with optimal tolerances determined by Spectronaut, and the correction factor was set to the system default [33,34]. Protein intensities were normalized by Spectronaut using a local normalization strategy. The total protein approach was employed for absolute protein level quantitation. Protein quantity was determined from raw intensity values using the formula: Protein (pmol/mg protein) = [(total intensity/(MW (g/mol) × total protein intensity)) × 10^9^.

### 2.10. Bioinformatic Analysis

Differentially expressed proteins were subjected to the ingenuity pathway analysis (IPA, release version 62089861, 2021; Qiagen, Germantown, MD, USA) to explore their potential biological functions [35]. The expression ratios of each protein to its control group were calculated based on their total protein concentrations. These values, along with the corresponding *p*-values (versus the control), were uploaded into the IPA to generate a database. Fisher’s exact test was used to identify significant canonical pathways, disease functions, and upstream regulators. The predictions of pathways, regulators, and disease functions were evaluated by comparing the direction of expression changes in the dataset to the expected expression patterns based on the available literature for each annotation. The z scores were assigned to indicate statistical significance.

### 2.11. Free Radical (DPPH) Scavenging Assay

The DPPH free radical scavenging capacity of CFA and CFB was measured as previously reported [36]. Briefly, a reaction solution containing 100 μL of serial diluted CFA or CFB, and the same volume of the DPPH solution was well mixed and then incubated for 30 min at room temperature in the dark. The absorbance of each sample was recorded at a wavelength of 517 nm using a SpectraMax M2 plate reader.

### 2.12. €-MGO Assay

The anti-glycation assay was performed with a reported method with minor modifications [37]. Briefly, MGO was used as a glycation reagent to induce the formation of €-AGEs. Every reaction mixture contained 600 μL of € (25 g/L), 100 μL of MGO (100 mM), and 800 μL of PBS buffer. A series of concentrations of samples (500 μL; ranging from 12.5 to 400 μM) were added to the €-MGO solution. After incubation at 37 °C for 7 days, the fluorescence intensity of each sample was analyzed by using a SpectraMax M2 plate reader with an excitation and emission wavelengths of 360 and 435 nm, respectively.

### 2.13. Apoptosis and Necrosis Assays

HaCaT cells were seeded in 6-well plates at 3 × 10^5^ cells per well and allowed to attach overnight. Then, samples were added and incubated for 4 h, followed by adding the MGO or GO solution for another 24 h. Cell culture medium was removed, and cells were washed with PBS for collecting cells. The cells pallet was resuspended in 100 μL of annexin-binding buffer containing 5 μL of Alexa Fluor TM 488 Annexin V and 1 μL of PI working solution. Samples were incubated for 15 min at room temperature and the stained cells were analyzed by flow cytometry (BD FACSCalibur, San Jose, CA, USA) [22].

### 2.14. Statistical Analysis

Data are presented as mean ± standard deviation (SD) from the replicates of three experiments. To generate a volcano plot using proteomics data, a significance threshold of 0.58, which corresponds to a 1.5-fold change, in conjunction with a *p*-value less than 0.05, was implemented. Statistical analyses were performed with GraphPad Prism 10 software by using ordinary one-way ANOVA analysis followed by Dunnett’s multiple comparisons testing. Significance for all tests was defined as * *p* < 0.05, ** *p* < 0.01, *** *p* < 0.001, **** *p* < 0.0001.

## 3. Results

### 3.1. RCSs Exacerbated Overall Cell Death but Did Not Affect Ferroptosis in Human Keratinocytes

**Figure 1:**
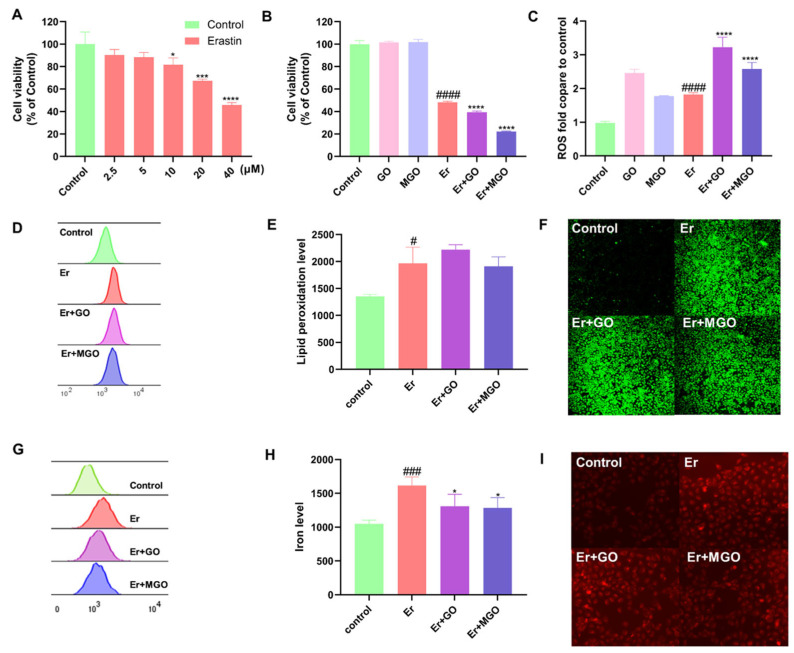
Biological evaluations of ferroptosis in HaCaT cells exposed to erastin, GO, MGO, and their combinations. (**A**) Effects of erastin (Er) (2.5–40 µM) on the cell viability of HaCaT cells. (**B**) Effects of stimulations with GO, MGO, and erastin alone as well as their combinations (Er + GO, and Er + MGO) on the cell viability of HaCaT cells. (**C**) Effects of MGO, GO, Er, Er + GO, and Er + MGO on the total production of ROS in HaCaT cells. (**D**) Flow cytometry assay for the lipid peroxide in HaCaT cells with the Liperfluo staining reagent. (**E**) Quantitative analysis of lipid peroxide detected by fluorescence signals. Data are shown as the averaged FITC values. (**F**) Images of lipid peroxidation in HaCaT cells exposed to erastin (20 μM) without or with GO or MGO (100 μM) for 24 h. (**G**) Flow cytometry analysis of intracellular iron using the FerroOrange staining reagent. (**H**) Quantitative analysis of Fe2+ detected by the fluorescence signals. Data are shown as the averaged PE values. (**I**) Images of FerroOrange stained HaCaT cells exposed to erastin (20 μM) without or with GO or MGO (100 μM) for 24 h. #### *p* < 0.0001, ### *p* < 0.001, # *p* < 0.05 as compared with the control group; * *p* < 0.05, *** *p* < 0.001 and **** *p* < 0.0001 as compared with the control and erastin-treated group.

A model of chemical-induced ferroptosis was established with human keratinocyte HaCaT cells. A ferroptosis inducer, namely, erastin (at 2.5, 5, 10, 20, and 40 µM), was used to insult HaCaT cells and reduced the cell viability to 90.2%, 88.4%, 81.8%, 67.4%, and 45.8%, respectively, suggesting that HaCaT cells are susceptible to ferroptosis (Figure 1A). Erastin-induced cytotoxicity in HaCaT cells was exacerbated when cells were exposed to a combination of erastin and RCSs including GO and MGO. Although GO or MGO (100 µM) alone did not induce significant cell death in HaCaT cells (cell viability > 98%; Figure S1), the combination of erastin and GO (Er + GO) or MGO (Er + MGO) led to aggravated cytotoxicity (cell viability of 39.8% and 22.2%, respectively) as compared to the erastin alone group (cell viability of 48.1%) (Figure 1B). The oxidative stress in HaCaT cells was elevated by the insult of erastin and RCSs. Erastin increased the level of cellular ROS by 1.83-fold as compared to the control group and its combination with GO or MGO elevated ROS production by 3.23- and 2.58-fold, respectively (Figure 1C). Increased cytotoxicity and ROS by the stimulation of erastin suggested that ferroptosis was induced in HaCaT cells. Although a similar trend was observed when HaCaT cells were treated with the combination of erastin and RCSs, it was not clear whether the cell death was attributed to ferroptosis. Thus, we further examined specific ferroptotic biomarkers, such as lipids peroxidation and cellular iron level, in HaCaT cells exposed to erastin vs. Er + GO or Er + MGO. A flow cytometry assay showed that erastin increased the level of lipid peroxidation in HaCaT cells (the fluorescence intensity increased from 1354.5 to 1967.3). The stimulation with Er + GO and Er + MGO also elevated the level of lipid peroxidation (as the fluorescence intensity increased to 1908.8 and 2219.0, respectively), which was not significant as compared to the erastin-treated cells (Figure 1D,E). This effect was supported by the confocal images of cells treated with erastin and erastin + RCSs (Figure 1F). Furthermore, a flow cytometry assay showed that cells treated with erastin, Er + GO, and Er + MGO had an increased iron level from 1048.7 to 1616.8, 1311.3, and 1286.3, respectively (Figure 1G,H). However, the iron level of cells treated with Er + GO and Er + MGO was slightly decreased as compared to the erastin-treated group (Figure 1H,I). Given that the exposure to Er + RCSs elevated cytotoxicity and ROS but not ferroptotic characteristics (i.e., lipid peroxidation and iron level) as compared to stimulation with erastin alone, it suggests that RCSs exacerbated overall cell death without promoting ferroptosis. It is possible that RCSs may have regulated the progress of cell death via specific molecular targets in a ferroptotic environment. This hypothesis was further explored by proteomic analyses.

### 3.2. Erastin and RCSs Altered Protein Expression in Human Keratinocytes

**Figure 2:**
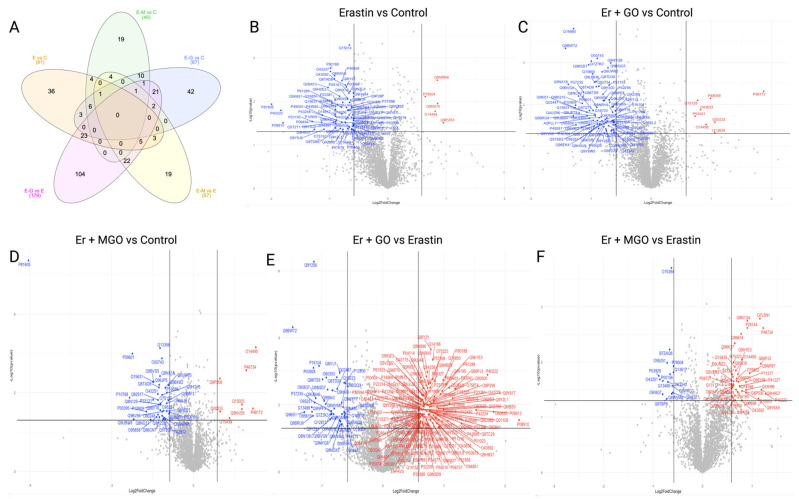
Effects on the number of proteins with significant changes in HaCaT cells exposed to erastin, GO, MGO, and their combinations by Venn diagrams and volcano plots. (**A**) Venn diagrams of proteins that were significantly enhanced or suppressed (*p* < 0.05) by at least 50% between the two comparing groups. (**B**–**F**) Volcano plots for the differential expression of proteins between different stimulation groups: Erastin vs. Control (**B**), Er + GO vs. Control (**C**), Er + MGO vs. Control (**D**), Er + GO vs. Erastin (**E**), and Er + MGO vs. Erastin (**F**). The volcano plots were generated using a cut-off value of 0.58 (1.5-fold change) and a *p*-value < 0.05. The x-axis represents the log2 fold change, and the y-axis represents the negative log10 *p*-value. Proteins with a log2 fold change above the cut-off and a *p*-value below the significance threshold are shown in red (upregulated) or blue (downregulated).

We examined the differences in the protein expression of HaCaT cells treated with erastin, RCSs (GO or MGO), and erastin combined with GO or MGO. Proteins extracted from HaCaT cells were sorted based on a threshold for significant changes (> 50%) with a *p*-value < 0.05, which resulted in the identification of a total of 3431 qualified proteins. A Venn diagram shows the number of proteins that meet the criteria with significant changes among all groups (Figure 2A). Erastin induced the expression changes of 81 proteins (5 upregulated and 76 downregulated) compared to the control group (Figure 2B). Stimulation with combinations of erastin and RCSs (GO or MGO) resulted in 87 and 46 protein expression changes, respectively, as compared to the control group (Figure 2C,D). In addition, the protein changes of cells treated with combinations of Er + GO and Er + MGO were compared with that of cells treated with erastin alone (Figure 2E,F). Notably, a number of protein changes (179) were identified in cells treated with Er + GO (vs. Er), including an increase of upregulated and downregulated proteins (134 vs. 45, respectively) (Figure 2F). Thus, the overall impact of a ferroptosis inducer and RCSs on protein expression in human keratinocyte HaCaT cells was captured at a proteomic scale. This provided further information from previously reported studies that only examined limited numbers of proteins with expression changes in keratinocytes induced by RCSs. For example, GO was reported to hinder the migration of keratinocytes by downregulating two specific proteins including epidermal growth factor receptor (EGFR) and its downstream effector Snai2 [38]. The identification of proteins with significant expression changes was achieved by employing the MS technique to measure a larger number of biomarkers (2096 proteins) that contributed to GO-induced premature cellular aging in keratinocytes [39]. Herein, we used LC-MS/MS with SWATH acquisition to achieve the identification of over 3000 proteins to depict a landscape of protein changes in multiple comparisons (i.e., erastin, GO, or MGO vs. the control group as well as Er + GO and Er + MGO vs. erastin alone). This enabled more advanced analyses on biomarkers that contributed to cell death induced by oxidative stressors (erastin, RCSs, and their combinations) and provided insights into their possible distinct mechanisms of action.

### 3.3. Canonical Pathways and Upstream Regulators in Ferroptotic HaCaT Cells

**Figure 3:**
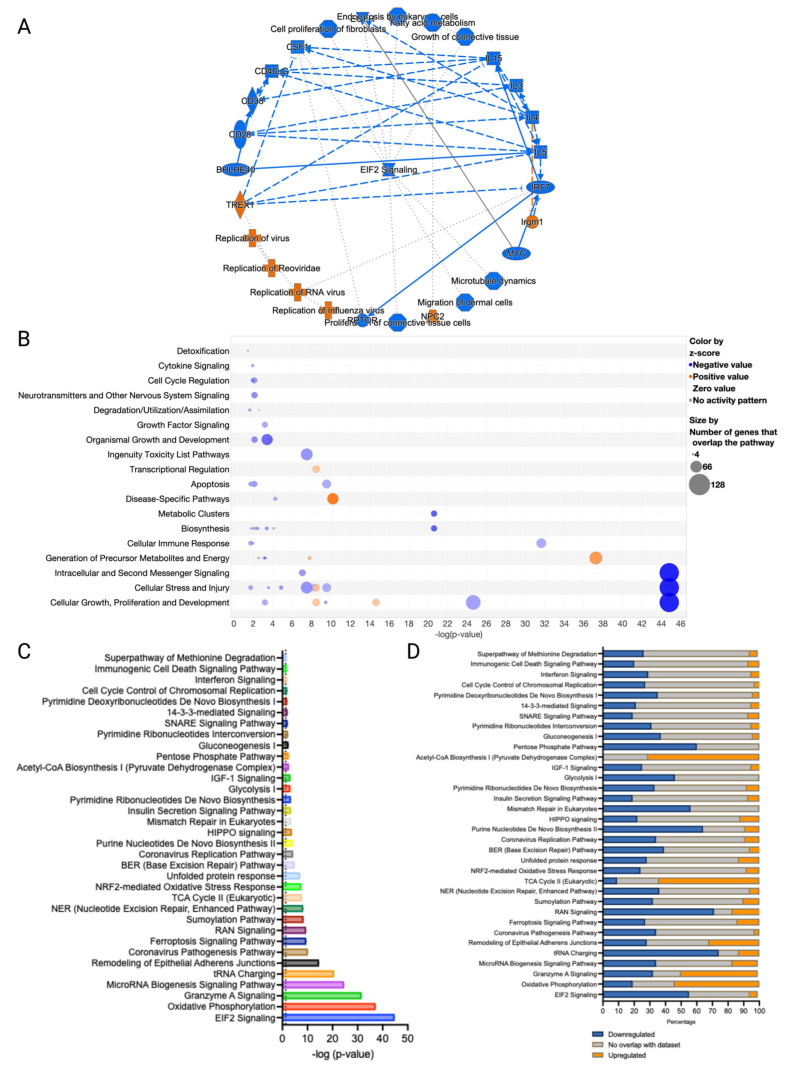
(**A**) Changes in canonical pathways and upstream regulators in the erastin-exposed group vs. control group. Canonical pathways and upstream regulators that were significantly activated (indicated by orange) or suppressed (indicated by blue) are shown. (**B**) Bubble chart summarizing proteins based on their −log(*p*-value). Proteins are color-coded by their z-score, with orange indicating a positive value and blue indicating a negative value. The size of each bubble corresponds to the number of genes that overlap the pathway. (**C**) Ingenuity canonical pathways of signaling pathways involved in erastin-exposed cells. Only pathways with a cut-off value of −log(*p*-value) > 1.3 and z-score > 2 are shown. (**D**) Horizontal stacked bar chart showing the percentage of downregulated, no overlap with the dataset, and upregulated proteins in the erastin group vs. control group.

In addition to the identification of proteins involved in ferroptosis in HaCaT cells, we conducted analyses of biomarkers including signaling pathways, key regulators, and cellular events in ferroptosis. Canonical pathway analysis revealed a molecular network of pathways involved in the activation of ferroptosis by erastin (Figure 3A). Erastin suppressed signaling pathways including eukaryotic initiation factor 2 (EIF2), dermal cell migration, microtubule dynamics, and fatty acid mechanisms. It also suppressed transcriptional regulators (MYC, IRF7, and BHLHE40), receptor proteins (CD28 and CD38), and cytokines (IL-3, IL-4, IL-5, IL-15, CD40LG, and CSF1). The major set of proteins that had a negative value were identified in the cellular events including cellular growth, cell proliferation and development, cellular stress and injury, and intracellular and second messenger signaling, whilst proteins involved in the generation of precursor metabolites and energy and disease-specific pathways had positive values (Figure 3B). The subsequent analysis showed the involvement of 166 canonical pathways, which were filtered into 25 distinct sets based on a cutoff of −log(*p*-value) > 1.3 and z-score > 2 (Figure 3C). In agreement with the pathway network analysis, the EIF2 signaling pathway was significantly suppressed (z-score = −5.578). We also filtered canonical pathways that were significantly altered by applying a cutoff of −log(*p*-value) > 1.3 and z-score > 2 (Figure 3C). Based on the ranking of the z-score, several prevalent canonical pathways, such as EIF2 signaling pathways [−log(*p*-value) = 44.8, z-score = −5.578], tRNA charging [−log(*p*-value) = 20.6, z-score = −4.116], and insulin secretion signaling pathways [−log(*p*-value) = 3.42, z-score = −3.732], were significantly suppressed. In contrast, pathways including coronavirus pathogenesis pathways [−log(*p*-value) = 10.2, z-score = 5.416], oxidative phosphorylation [−log(*p*-value) = 37.2, z-score = 4.163], and HIPPO signaling [−log(*p*-value) = 3.77, z-score = 3.3] were activated. To validate these findings, we compared our dataset with the IPA database for the number of proteins that were down- or upregulated in these identified pathways (Figure 3D). For instance, in the EIF signaling pathways, we identified 117/221 (55%) downregulated proteins, 11/211 (5%) upregulated proteins, and 83/211 (40%) non-overlapping proteins in the IPA dataset. Similarly, in the oxidative phosphorylation pathway, we identified 21/109 (19%) downregulated proteins, 59/109 (54%) upregulated proteins, and 29/109 (27%) non-overlapping proteins in the dataset.

### 3.4. GO and MGO Had Distinct Influences on Pathways Involved in Erastin-Induced Cell Death

**Figure 4:**
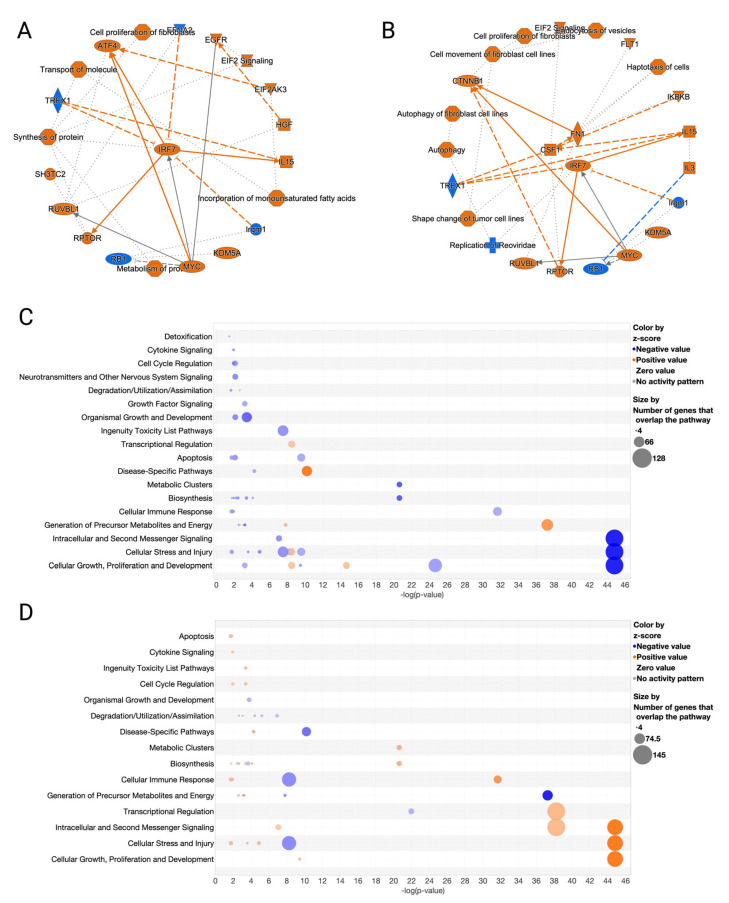
Changes in canonical pathways and upstream regulators in the (**A**) Er + GO-exposed group or (**B**) the Er + MGO-exposed group vs. the erastin group. Canonical pathways and upstream regulators that were significantly activated (indicated by orange) or suppressed (indicated by blue) are shown. Bubble chart summarizing proteins changes in (**C**) Er + GO- and (**D**) Er + MGO-exposed group based on their −log(*p*-value). Proteins are color-coded by their z-score, with orange indicating a positive value and blue indicating a negative value. The size of each bubble corresponds to the number of genes that overlap the pathway.

**Figure 5:**
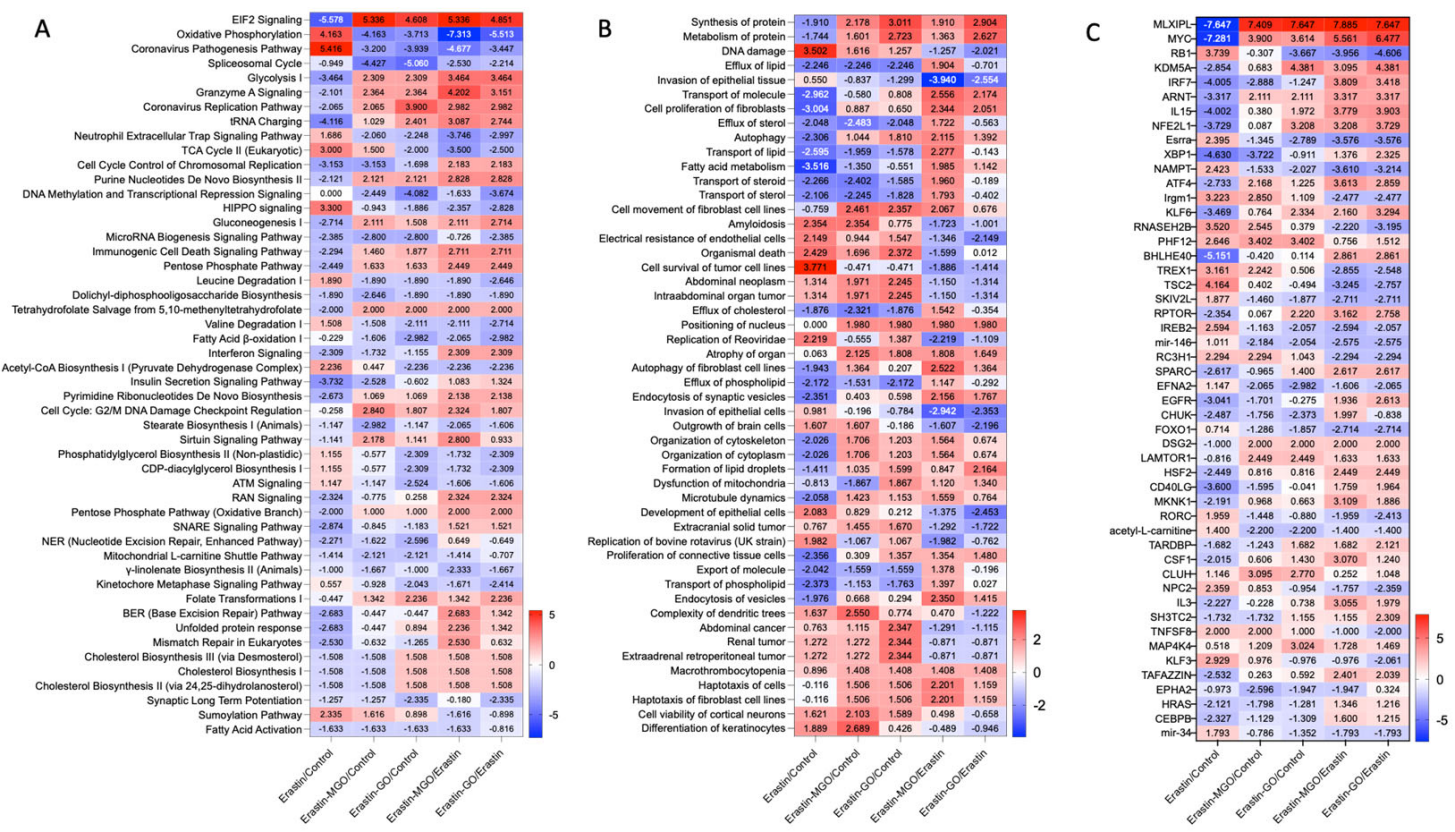
Comparison of the canonical pathways, diseases and functions, and upstream regulators among all the groups to identify significant differences in cells exposed to different stimulations. (**A**) Comparison analysis was performed using a cut-off value of −log(*p*-value) > 1.3 and z-score > 2. The heatmap displays the results of this analysis, with numbers indicating the z-score. Panel (**A**) shows the top 50 canonical pathways selected from 166 signaling pathways. Panel (**B**) shows the top 50 diseases and functions selected from 347 options. Panel (**C**) shows the top 50 upstream regulators selected from 209 options.

Our biological evaluations showed that the addition of GO or MGO to erastin can elevate cellular oxidative stress and exacerbate the overall cell death in HaCaT cells. It is unclear whether the Er + GO- and Er + MGO-induced cell death pathways were identical to that of erastin-induced ferroptosis. Herein, we investigated the impact of GO and MGO on the molecular network of erastin-induced cell death. Notably, distinct changes in the signaling pathways, regulators, and cellular events of cells treated with Er + GO were observed in comparison to that of cells insulted with erastin alone. For instance, the EIF2 signaling pathway, which was suppressed in erastin-treated cells, was significantly activated in cells exposed to Er + GO (Figure 4A). We also observed the significant activation of transcription factors (IRF7, ATF4, RUVBL1, MYC, and KMD5A), cytoplasmic proteins (RPTOR and EIF2AK3), receptor (EGFR), extracellular protein (HGF), and cytokine (IL-15). Similar effects were observed in pathways of cellular functions including the incorporation of monounsaturated fatty acids, protein synthesis, and molecule transport. Treatment with Er + GO significantly suppressed several proteins including RB1, Irgm1, TREX1, and EFNA2 as compared to cells treated with erastin. In the subsequent analysis, identified pathways were classified into 25 categories, revealing a significant increase in the EIF2 signaling pathway, whilst a suppression of oxidative phosphorylation in Er + GO exposed cells (Figure 4B). This supported that the Er + GO treatment led to distinct alterations in cellular signaling pathways as compared to the stimulation of erastin alone. A comprehensive analysis was performed to identify the canonical pathways that were activated or suppressed upon Er + GO treatment. Similar analyses were performed for the Er + MGO group in comparison to the erastin group (Figure 5). A similar activation/suppression pattern as the Er + MGO and Er + GO shared overlapped changes in the signaling pathways. Canonical pathways including the EIF2 signaling pathway and fibroblast cell proliferation, along with transcription factors (IRF7, MYC, RUVBL1, RB1, and CTNNB1), cytoplasmic protein (RPTOR), and cytokine (IL-15) exhibited similar alterations in both Er + MGO and Er + GO exposed cells. In addition, the Er + MGO group had activated pathways of FN1, CSF1, IKBKB, and IL-13 as well as promoted cell haptotaxis, endocytosis of vesicles, cell movement, and autophagy of fibroblast in comparison to that of the erastin-treated cells. We also investigated the alterations by Er + MGO treatment in signaling pathways in 25 distinct categories (Figure 5B). Similar to the Er + GO group, stimulation with Er + MGO altered pathways including EIF2, oxidative phosphorylation, and granzyme A.

It was noted that GO and MGO altered the signaling pathways of EIF2, which is a protein that is responsible for the initiation of eukaryotic translation. The EIF2 protein is a heterotrimer comprising three subunits including the alpha subunit (EIF2-α), the beta subunit (EIF2-β), and the gamma subunit (EIF2-γ) [40]. The subunits of this protein mediate important cell survival processes by a consequence of phosphorylation and dephosphorylation. Upon phosphorylation, EIF2 exhibits a strong binding affinity towards EIF2-β, resulting in the complete inhibition of translation [41]. To alleviate the translational elongation of essential transcriptional factors, a cellular stress response protein (GADD34) can dephosphorylate EIF2-α [42]. The phosphorylation of EIF2-α triggers apoptosis and further leads to the termination of the global translation of proteins. This provides a possible mechanism for the observation of elevated ferroptosis and exacerbated overall cell death in cells treated with Er + GO and Er + MGO. This was also in agreement with our proteomics analyses which showed that suppression of EIF2 by erastin was counteracted by the combinations of erastin and GO or MGO.

### 3.5. Erastin and RCSs Had Distinct Changes in Canonical Pathways and Upstream Regulators

To further gain insights into the impact of ferroptosis (induced by erastin) and overall cell death (induced by GO, MGO, Er + GO, and Er + MGO) in HaCaT cells, we analyzed changes in canonical pathways, diseases and functions, and upstream regulators for all groups (i.e., erastin vs. control, Er + GO vs. control, Er + MGO vs. control, Er + GO vs. erastin, and Er + MGO vs. erastin). We established a dataset consisting of 3430 protein IDs and identified 166 canonical signaling pathways, 347 diseases and functions, and 209 upstream regulators using the IPA tool. We then selected the top 50 targets from each category for further analysis. As shown in Figure 5A, there was significant suppression of eukaryotic initiation factor 2 (EIF2) signaling, glycolysis I, granzyme A signaling, coronavirus replication pathways, and cell cycle control of chromosomal replication with a z-score of −5.578, −3.454, −2.101, −2.065, and −3.153, respectively, in erastin-exposed cells as compared to the normal cells. These results suggest that erastin affected several critical pathways involved in functional events including cellular metabolism, immune response, viral replication, and cell cycle regulation. In contrast, GO or MGO reversed the suppression of the aforementioned pathways as evidenced in the positive z-scores of EIF2 signaling, glycolysis I, granzyme A signaling, coronavirus replication pathways, and cell cycle control of chromosomal replication (4.851, 3.464, 3.151, 2.982, and 2.183, respectively). Similarly, canonical pathways including oxidative phosphorylation (z-score = 4.153), coronavirus pathogenesis pathway (z-score = 5.416), TCA cycle II (z-score = 3.0), HIPPO signaling (z-score = 3.30), and acetyl-CoA biosynthesis I (z-score = 2.236) were activated in the cells exposed to erastin. These activations were counteracted by the treatment with GO and MGO, which suppressed these pathways with negative z-scores of oxidative phosphorylation (−5.513 and −7.313), coronavirus pathogenesis pathway (−3.447 and −4.677), TCA cycle II (−2.5 and −3.5), HIPPO signaling (−2.828 and −2.357), and acetyl-CoA biosynthesis I (−2.236 and −2.236), respectively. We also noted several signaling pathways that were only affected by GO or MGO. For instance, erastin did not affect DNA methylation and transcriptional repression signaling (z-score = 0), which was suppressed by GO and MGO, with negative z-scores of −1.633 and −3.674, respectively.

**Figure 6:**
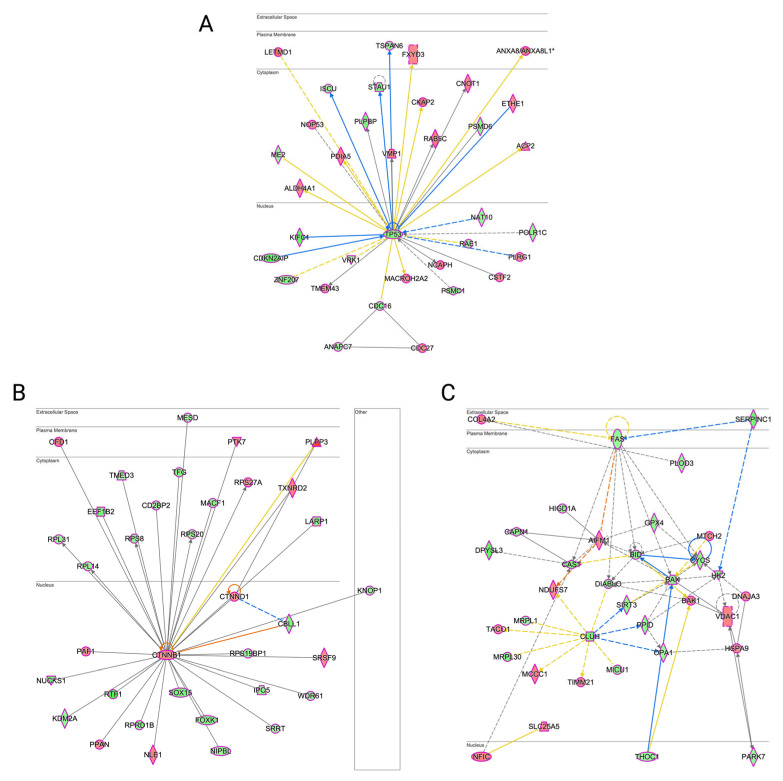
Network diagram depicting the protein targets involved in the network. Red nodes represent upregulated molecules and green nodes represent downregulated molecules. (**A**) Molecule network associated with cell cycle, cell death and survival, and embryonic development in each group. The presence of an overlap between ANXA8 and ANXA8L in the networks was indicated by the use of the asterisk (*) (**B**) Molecule network associated with cell death and survival, DNA replication, recombination, and repair in each group. (**C**) Molecule network associated with cell death and survival, embryonic development, and organismal injury and abnormalities in each group.

Furthermore, changes of disease and functions by erastin and RCSs were analyzed. Although our cellular model with HaCaT cells may not represent certain disease conditions such as abdominal cancer, renal tumor, abdominal neoplasm, and extracellular solid tumors, proteomics analysis identified biological functions that were associated with cellular physiology and homeostasis. As shown in Figure 5B, erastin resulted in DNA damage (z-score = 3.502), the cell survival of tumor cells (z-score = 3.771), and organismal death (z-score = 3.429), whereas the presence of GO or MGO ameliorated the activation of these functions with negative z-scores for DNA damage (−2.021 and −1.257), the cell survival of tumor cells (−1.414 and −1.886), and a deducted z-score in organismal death (0.012 and −1.599) in the Er + GO and Er + MGO groups, respectively. Moreover, erastin suppressed several functions including fatty acid metabolism (z-score = −3.516), molecule transport (z-score = −3.516), steroid metabolism (z-score = −2.266), sterol metabolism (z-score = −2.106), and phospholipid efflux (z-score = −2.172). Again, the presence of GO or MGO counteracted erastin-induced suppression as evidenced by the negative or reduction in z-scores for fatty acid metabolism (1.412 and 1.985), molecule transport 2.174 and 2.556), steroid metabolism (−0.189 and 1.960), sterol metabolism (−0.402 and 1.793), and phospholipid efflux (−0.701 and 1.904), respectively. We further analyzed a set of 209 regulators and identified the top 50 upstream regulators. Among them, two transcription factors, namely, Max-like protein X interacting protein-like (MLXIPL) and MYC, are known for their role in the regulation of the EIF2 signaling pathway [43]. Notably, MLXIPL and MYC were found to be significantly suppressed by erastin with a z-score of −7.647 and −7.281, respectively (Figure 5C). Erastin-induced suppression of MLXIPL and MYC was activated by GO (with a z-score of 7.647 and 7.409) and MGO (with a z-score of 6.477 and 5.561), respectively. Apart from suppressing regulators, erastin-induced ferroptosis activated several regulators. Retinoblastoma protein (RB1; a negative regulator of the cell cycle) encoded protein can stabilize constitutive heterochromatin to maintain overall chromatin structure [44]. In ferroptotic cells induced by erastin, RB1 was activated with a z-score of 3.739. Exposure to GO or MGO suppressed the activation of RB1 with a z-score of −4.606 and −3.956, respectively. A similar pattern was also observed that several regulators including KDM5A, IRF7, ARNT, IL15, NEF2L1, BHLHE40, EGFR, CD40LG, TAFAZZIN, KLF6, and ATF4 were suppressed in erastin-induced ferroptotic cells but the suppressions were counteracted by GO and MG as evidenced by their z-scores (Figure 6C).

Several regulators identified here have been reported to be mediators for ferroptosis. For instance, MLXIPL, encoding the carbohydrate-response element-binding protein (ChREBP), is a basic helix–loop–helix leucine zipper transcription factor of the Myc/Max/Mad superfamily [45]. It forms a heterodimeric complex and binds to carbohydrate response element motifs in the promoters of triglyceride synthesis genes, leading to the metabolism of excess glucose to fatty acids via the acetyl-CoA route [46]. MLXIPL also mediates the carbohydrate induction of lipogenesis and lipid homeostasis, which collectively regulate the metabolism of carbohydrates and lipids in cells [47,48]. Given that erastin and RCSs are known inducers of lipid peroxidation and metabolites of glucose, respectively, it is not surprising that MLXIPL was identified as a key regulator in erastin-induced ferroptosis in the presence of RCSs. Notably, MYC, a regulatory gene for cell proliferation, differentiation, and apoptosis, was suppressed in erastin-exposed cells, which was in agreement with its mediation of ferroptosis in reported studies [49,50]. MYC also serves as a regulator in cellular glycolysis by activating lactate dehydrogenase, glucose transporter 1, and hexokinase 2, which plays a crucial role in the detoxification of GO and MGO [51]. Further studies with functional assays are warranted to confirm the regulatory roles of targets identified from our proteomics analyses.

### 3.6. Erastin-Induced Ferroptosis and RCSs-Induced Cell Death Had a Different Impact on Molecular Networks Related to Cell Death and Survival

Networks related to cell cycle, cell death and survival, and embryonic development were constructed with molecular targets for diseases and functions (Figure 6A). It was revealed that TP53, a central transcriptional factor regulating various programmed cell deaths including apoptosis, autophagic cell death, pyroptosis, and ferroptosis [52], was downregulated by 1.262-fold in erastin-induced ferroptosis. This downregulation was counteracted by GO or MGO, which increased the expression of TP53 with fold changes of 1.274 and 1.229, respectively. In addition, genes encoding proteins that can bind to TP53, such as CDKN2AIP, were suppressed in the erastin-induced ferroptosis, whilst MGO counteracted the suppression. Erastin-induced ferroptosis also increased the expression of the gene iron–sulfur cluster assembly enzyme (ISCU), which is a protein associated with the iron homeostasis signaling pathway [53]. Suppressed ISCU expression was reversed to 1.004-fold in HaCaT cells exposed to Er + MGO. In the network related to ‘cell death and survival, DNA replication, recombination and repair, and gene expression’, cyclin-dependent kinase inhibitor 2A gene [54] (CTNNB1; a transcription factor located at the center of the network) was found to be increased by 1.070-fold in erastin-induced ferroptosis cells (Figure 6B). Notably, CTNNB1 encodes a protein that is part of the adherent junction complex responsible for regulating cell growth and adhesion in epithelial cell layers. Increased expression of CTNNB1 in ferroptotic cells was suppressed by GO to 1.057-fold. Additionally, the transcription factor CTNND1 was also activated to 1.067-fold in erastin-exposed HaCaT cells, whilst its expression was suppressed to −1.088- and −1.045-fold, by GO or MGO, respectively. This molecular network suggested that CTNNB1 and CTNND1 played critical roles in the events of DNA replication, recombination, and repair as well as cell death and survival. We also analyzed a network related to cell death and survival, embryonic development, and organismal injury and abnormalities (Figure 6C). It revealed that the expression of FAS, a member of the TNF-receptor superfamily with a crucial role in regulating programmed cell death physiologically, was suppressed in ferroptotic cells exposed to erastin. Stimulation of GO and MGO had opposing effects on the expression, in which FAS expression was activated by MGO but suppressed by GO. In addition, two genes including BAX and CLUH were identified at the center of the network. BAX is a well-known apoptosis regulator that forms a heterodimer with BCL2 to activate apoptosis [55]. BAX was suppressed in erastin-induced ferroptotic cells, while Er + GO increased BAX expression and Er + MGO decreased BAX expression. CLUH (a gene that encodes clustered mitochondria homolog protein) was also decreased in erastin-induced ferroptotic cells. The presence of GO and MGO resulted in increased CLUH expression as compared to that of the erastin-exposed cells.

Based on the analyses of networks related to cell death and survival, embryonic development, and organismal injury and abnormalities, a series of genes were identified as critical targets that mediated erastin-induced ferroptosis. These findings were supported by previously reported studies. For instance, TP53 was reported to bind to the p53-responsive element in the promoter region of a cystine/glutamate transporter (encoded by SLC7A11). This interaction leads to the suppressed TP53 expression, resulting in ferroptosis by enhancing the sensitivity of cells to erastin [56]. Moreover, genes identified from the network analysis, such as ISCU [8], STAU1 [57], and PDIA5 [58], have been reported to regulate iron uptake, metabolism, and storage, resulting in the susceptibility of cells to ferroptosis. Similarly, the links between ferroptosis and genes identified from the networks related to cell death and survival (Figure 6) are supported by reported studies. For instance, BAX can directly mediate the mitochondrial pathway, which plays a critical role in the crosstalk between ferroptosis and apoptosis [59,60]. Additionally, BAX’s expression was reported to be mediated by MGO, which resulted in the progression of apoptosis in murine neuroblastoma cells (Neuro-2A) [61]. These identified genes provided possible mechanisms for the findings from our biological assays (Figure 1), in which erastin induced ferroptosis in HaCaT cells and its combination with RCSs exacerbated the cell death in a manner that was independent of ferroptosis. Although the proteomics study was able to provide information on the overall changes of proteins in cells exposed to erastin, RCSs, and their combinations, further investigations are warranted to validate and confirm targets identified from the proteomics-based network (Figure S2). Additional biological and/or biophysical assays are required to examine the changes in target proteins at structural and functional levels. Nevertheless, findings from our biological assays and proteomics analyses supported that oxidative stress is a major factor contributing to ferroptosis (by erastin) and general cell death (by RCSs) in human keratinocytes. In summary, data from the proteomics analysis showed that RCSs (when combined with erastin) affected a panel of cell death-related molecular targets, which played critical roles in the maintenance of cellular redox homeostasis. This tentative mechanism is supported by studies showing that RCSs can affect protein targets such as aldehyde dehydrogenases, aldo-keto reductases, carbonyl reductase, and glutathione S-transferases [62], which also contribute to the mediation of ferroptosis. Apart from redox homeostasis-related proteins, the cellular detoxification system consisting of enzymes (e.g., glyoxalase) that produce D-lactate and glutathione from MGO metabolites may also play a role in RCSs-induced cell death [63]. Given that glutathione is a critical mediator for ferroptosis, it is possible that mechanisms involved in the detoxification of RCSs also contribute to the overall cellular impact of RCSs and ferroptosis. Our data demonstrated that the crosstalk between ferroptosis and general cell death by erastin and RCSs were mediated via distinct pathways and molecular targets. This is particularly important in the context of developing therapeutic interventions for diabetic complications, given that cellular reactive carbonyl stress and ferroptosis-driven cellular damage are both prevalent in diabetes pathological conditions. Next, we sought to evaluate dietary polyphenols that can rescue HaCaT cells from RCSs + erastin-induced cytotoxicity.

### 3.7. CFA and CFB Alleviated Erastin-Induced Cytotoxicity in HaCaT Cells by Reducing Cellular Lipid Peroxidation

We next sought to study the cytoprotective effects of a library of dietary natural products against RCSs + erastin-induced cell death. It was noted that a chemotype of natural products, namely, flavonoids, showed promising antioxidant and cytoprotective effects against ferroptosis in a preliminary screening study. This was not surprising given that flavonoids from dietary resources are reported to exert various anti-aging activities including cytoprotective, antioxidant, anti-glycation, and anti-inflammatory effects [64,65]. Furthermore, a group of flavonoids with unique chemical structures (with a prenyl or geranyl moiety on the flavonoid skeleton) from the cannabis plant (*C. sativa*) showed promising cytoprotective effects. Although cannabis compounds are reported to show anti-aging effects in skin models [66], most of the research efforts have been directed to investigate the cannabinoid-type of phytochemicals. However, the anti-aging effects of other constituents of cannabis, such as flavonoids, remain unclear. Therefore, based on our preliminary data and literature reports, we further selected two representative flavonoids (i.e., CFA and CFB) from cannabis as chemical probes to investigate the potential anti-skin-aging effects in a cell death model with RCSs and erastin.

**Figure 7:**
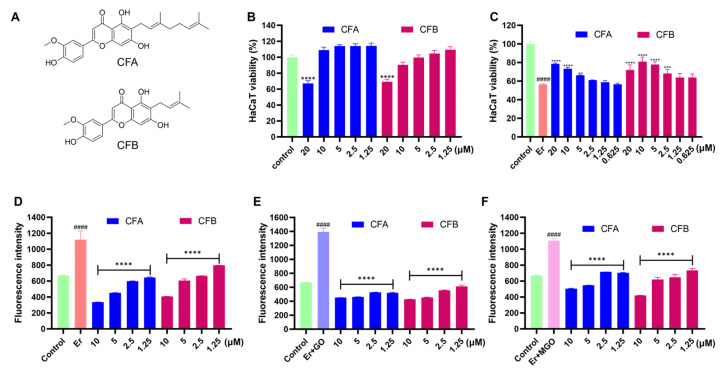
(**A**) The chemical structure of CFA and CFB. (**B**) Effects of CFA and CFB on the cell viability of HaCaT cells without the presence of erastin. Effects of CFA and CFB on the cell viability (**C**) and lipid peroxidation (**D**) of HaCaT cells exposed to erastin. Effects of CFA and CFB on lipid peroxidation of HaCaT cells exposed to the combinations of Er + MGO (**E**) and Er + GO (**F**). #### *p* < 0.0001 as compared with the control group, ** *p* < 0.01, *** *p* < 0.001, **** *p* < 0.0001 as compared with Er, Er + MGO, or Er + GO group.

We first assessed the cytoprotective effects of CFA and CFB against cytotoxicity induced by erastin alone in HaCaT cells. CFA and CFB showed no significant cytotoxicity at concentrations ranging from 1.25 to 10 μM and mild toxicity at the higher concentration (20 μM) (Figure 7B). Then, CFA and CFB at concentrations of 0.63, 1.25, 2.5, 5, 10, and 20 μM reduced erastin-induced cytotoxicity by increasing the cell viability to 56.6%, 58.9%, 61.0%, 66.3%, 73.3%, and 78.6%, and to 63.8%, 63.9%, 68.1%, 77.9%, 81.1%, and 72.0%, respectively (Figure 7C). Next, we evaluated the effects of CFA and CFB on cellular lipid peroxidation, which is a biomarker of ferroptosis. CFA and CFB (at concentrations of 1.25, 2.5, 5, and 10 μM) decreased lipid peroxidation by 42.3%, 46.5%, 59.7%, and 70.2%, and by 28.8, 40.7, 45.9, and 63.6%, respectively (Figure 7D). We further evaluated the effects of CFA and CFB on the lipid peroxidation induced by the combinations of erastin + MGO (Figure 7E) and erastin + GO (Figure 7F). CFA and CFB (1.25–10 μM) reduced lipid peroxidation by 36.6% to 54.5% and by 33.8 to 62.0%, respectively, in the erastin + MGO model, as well as by 62.7% to 67.6% and by 56.1% to 69.3%, respectively, in the erastin + GO group.

### 3.8. CFA and CFB Ameliorated Cytotoxicity Induced by Er + GO and Er + MGO in HaCaT Cells

**Figure 8:**
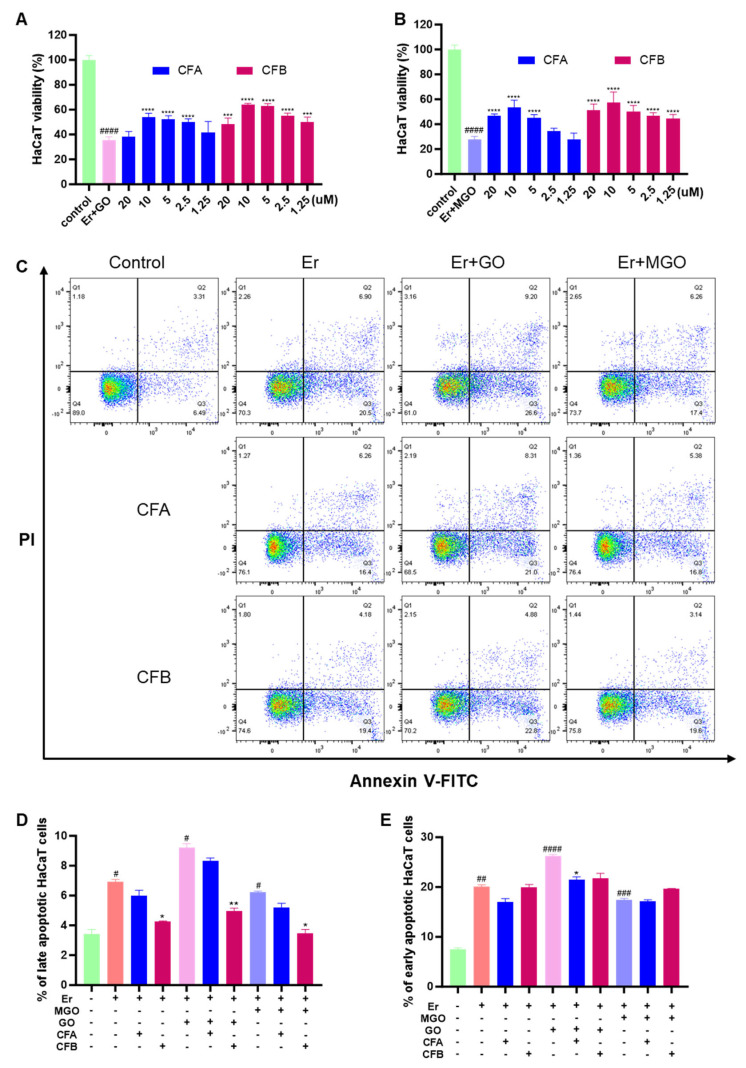
The effects of CFA and CFB on Er + GO (**A**) and Er + MGO (**B**) induced the cell viability of HaCaT cells. (**C**) Flow cytometry dot plots of Er-, Er + GO-, and Er + MGO-induced apoptosis in HaCaT cells. Changes in the percentage of late (**D**) and early (**E**) apoptosis stage in HaCaT cells. # *p* < 0.05, ## *p* < 0.01, ### *p* < 0.001, #### *p* < 0.0001 as compared with the control group, * *p* < 0.05, ** *p* < 0.01, *** *p* < 0.001, **** *p* < 0.0001 as compared with Er, Er + MGO, or Er + GO group.

Given that the combination of erastin and RCSs did not exacerbate ferroptosis but increased the overall cell death of HaCaT cells, we further evaluated whether CFA and CFB can counteract the cytotoxic effects of erastin + GO and erastin + MGO. As shown in Figure 8A, CFA and CFB (both at concentrations of 0.63, 1.25, 2.5, 5, 10, and 20 μM) decreased Er + GO-induced cytotoxicity by increasing the viabilities of HaCaT cells to 47.4, 48.9, 51.2 62.8, and 46.5%, and 49.7 55.2, 62.9, 63.9, and 48.2%. Similarly, CFA and CFB restored the cell viability to 22.3, 36.9, 48.7, 55.8, and 47.7%, and 41.9 50.3, 52.2, 54.1, 38.4%, respectively, in the model group of erastin + MGO. To further study the effects of CFA and CFB on the cell death of HaCaT cells induced by erastin and erastin with RCSs, we conducted flow cytometric assays to characterize apoptosis in HaCaT cells (Figure 8C). HaCaT cells exposed to Er, Er + GO, and Er + MGO showed increased late apoptotic populations (cells that stained PI^+^ and Annexin^+^; 6.9%, 9.2%, and 6.3%, respectively), as compared to the control group (3.4%). This effect was counteracted by CFA and CFB (at 10 μM), which decreased the populations of the late apoptotic cells to 6.0%, 8.3%, 5.2%, and to 4.2%, 4.9%, and 3.5%, respectively (Figure 8C). CFA and CFB also counteracted Er-, Er + MGO-, and Er + GO-induced early-stage apoptosis in HaCaT cells. HaCaT cells had an early apoptotic population of 20.0%, 26.2%, and 17.4% when they were exposed to the insult of Er, Er + MGO-, and Er + GO, respectively, whereas it was 7.5% in the control group. CFA and CFB (at 10 μM) decreased the early apoptosis to 17.1%, 21.5%, and 17.1%, and to 19.9%, 21.8%, and 19.6%, respectively.

### 3.9. CFA and CFB Scavenged Free Radicals and Inhibited the Formation of AGEs

**Figure 9:**
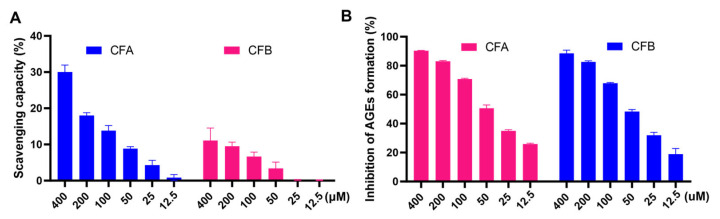
The effects of CFA and CFB on the free radical scavenging capacity (**A**) and the formation of MGO-induced AGEs formation (**B**).

Antioxidant capacity is identified as a key mechanism that involves in the anti-ferroptosis [67] and anti-apoptosis [68] effects of dietary natural products. Thus, we conducted two antioxidant assays including the DPPH free radical scavenging and AGEs inhibition assay. In the DPPH assay, CFA (concentrations ranged from 12.5 to 400 μM) showed an antioxidant capacity with a free radicals scavenging rate of 0.8–26.8%, respectively. CFB showed a weaker antioxidant capacity with a scavenging rate of 0.0% to 11.1% at 12.5–400 μM, respectively. In the AGEs inhibition assay, both CFA and CFB showed promising anti-glycation effects against MGO-induced AGEs formation of 25.9%, 35.0%, 50.5%, 70.7%, 83.1%, and 90.4%, and by 19.0%, 31.9%, 48.3%, 67.9%, 82.6%, and 88.6%, at the concentrations of 12.5, 25, 50, 100, 200, and 400 μM, respectively (Figure 9).

It is possible that the cytoprotective effects of CFA and CFB against ferroptosis are associated with their antioxidant capacity. This is supported by reported studies showing that flavonoids including quercetin [69] and butein [70] with free radical scavenging effects are inhibitors of ferroptosis. However, given that CFA and CFB only showed a moderate antioxidant capacity in the DPPH assay, it is likely that their anti-ferroptosis effects are mediated by the regulation of cellular targets. Thus, further studies are warranted to further examine whether CFA and CFB alleviated ferroptosis via protein targets identified in the proteomics analysis. Notably, CFA and CFB showed promising anti-glycation activity, which is possibly contributed to their protective effects against MGO-induced toxicity in HaCaT cells. This is in agreement with our previously reported data showing that dietary polyphenols from red maple and pomegranate can inhibit MGO-induced AGEs formation and ameliorate MGO-induced cytotoxicity [4,24].

Although CFA and CFB showed promising cytoprotective effects in the skin cells-based model, further studies using a combination of in vitro and in vivo models are warranted to investigate the potential anti-aging effects of cannflavins. For instance, the skin permeability of CFA and CFB remains unclear and studies using various techniques including a computational approach, skin-membrane assays (e.g., parallel artificial membrane permeability assay), ex vivo methods (Franz cell test), and in vivo models are warranted to understand the physiological impact of cannflavins on skin-aging. Findings from these studies are important for properly designed human clinical studies, which are critical for the development of cannflavins as bioactive ingredients for nutraceutical and/or cosmeceutical applications.

## 4. Conclusions

In summary, we conducted cell-based assays to characterize erastin-induced ferroptosis in HaCaT cells. In addition, we showed that RCSs including GO and MGO did not aggravate erastin-induced ferroptosis but exacerbated the overall cytotoxicity of HaCaT cells. The distinct toxic effects of erastin and RCSs on HaCaT cells were supported by scrutinizing specific ferroptotic biomarkers including the ROS production, lipid peroxidation, and cellular iron level. In addition, a SWATH-based proteomic study was conducted to identify proteins that were involved in erastin-induced ferroptosis and RCSs-induced cell death at a scale of over 3000 proteins, which captured a landscape of protein changes in HaCaT cells by comparisons of multiple stimulation groups. Furthermore, DIA-based bioinformatic analyses revealed tentative molecular targets including canonical pathways, upstream regulators, and gene networks that contributed to the biological effects of erastin and RCSs in HaCaT cells. To date, this is the first study combining bioassays and proteomics to investigate the molecular targets of ferroptosis in the context of RCSs-induced toxicity in HaCaT cells. Using this cell death model, we further evaluated the cytoprotective effects of two dietary polyphenols cannflavins. CFA and CFB protected HaCaT cells from ferroptosis induced by erastin and apoptosis induced by the combinations of erastin and RCSs. The cytoprotective effects of CFA and CFB were associated with their antioxidant activities including reducing lipid peroxidation, free radical scavenging, and the inhibition of AGEs formation. Together, findings from the current study provided valuable insights into the proteomic landscape of ferroptosis in HaCaT cells, which is useful as a model to evaluate the cytoprotective effects of dietary polyphenols for their potential therapeutic applications for skin aging.

## Data Availability

All proteomic datasets analyzed or generated during the study are publicly archived. The raw mass spectrometry data, along with the quantification results, for this study, have been archived in the MassIVE repository (https://massive.ucsd.edu/ProteoSAFe/static/massive.jsp) under the project name “MassIVE MSV000093197”. The corresponding FTP download links are available ftp://massive.ucsd.edu/v02/MSV000093197.

## Supplementary Materials

The following supporting information can be downloaded at: https://www.mdpi.com/article/10.3390/nu15214565/s1, Data on the cell viability of HaCaT cells exposed to different concentrations of RCSs (Figure S1) and the networks related to cell death and survival among all the stimulation groups (Figure S2) are provided in the Supplementary Materials.

## Abbreviations

AGEs	advanced glycation endproducts
ARNT	aryl hydrocarbon receptor nuclear translocator
ATF4	activating transcription factor 4
BHLHE40	basic helix–loop–helix family member e40
CD	cluster of differentiation
CD40LG	cd40 ligand
CDKN2AIP	CDKN2A interacting protein
ChREBP	carbohydrate-response element-binding protein
CSF1	colony stimulating factor 1
CTNNB1	catenin beta 1
DIA	data-independent acquisition
DOC	sodium deoxycholate
DPPH	2,2-diphenyl-1-picrylhydrazyl
EFNA2	ephrin A2
EGFR	epidermal growth factor receptor
EIF2	eukaryotic initiation factor 2
EIF2AK3	eukaryotic translation initiation factor 2 alpha kinase 3
FDR	false discovery rate
FN1	fibronectin 1
GADD34	growth arrest and DNA damage-inducible protein
GO	glyoxal
GOLD	glyoxal-lysine dimer
GPX4	glutathione peroxidase 4
GSH	glutathione
HGF	hepatocyte growth factor
HSA	human serum albumin
IAA	Iodoacetamide
IKBKB	inhibitor of kappa light polypeptide gene enhancer in B-cells, kinase beta
IL	interleukin
IRF7	interferon regulatory factor 7
Irgm1	immunity-related GTPase M 1
ISCU	iron-sulfur cluster assembly enzyme
KDM5A	lysine demethylase 5A
KLF6	Kruppel-like factor 6
MGO	methylglyoxal
MLXIPL	Max-like protein X interacting protein-like
MOLD	methylglyoxal-lysine dimer
MS	mass spectra
NEF2L1	nuclear factor erythroid 2-related factor 1
PSM	peptide-spectrum match
RB1	retinoblastoma 1
RCSs	reactive carbonyl species
RPTOR	regulatory-associated protein of mTOR
RUVBL1	RuvB-like 1
SLC7A11	cystine/glutamate transporter
SWATH	sequential window acquisition of all theoretical
TP53	tumor protein p53
TREX1	three prime repair exonuclease 1
