# Supplementary material for: Cannflavins A and B with Anti-Ferroptosis, Anti-Glycation, and Antioxidant Activities Protect Human Keratinocytes in a Cell Death Model with Erastin and Reactive Carbonyl Species
Source: Nutrients. 2023 Oct 27;15(21):4565. doi: 10.3390/nu15214565 (PMC10650133; doi:10.3390/nu15214565)
Supplement: Supplementary file 1 [file nutrients-15-04565-s001.zip › nutrients-2647956-supplementary.pdf]

SUPPLEMENTAL MATERIALS

Cannflavins A and B with Anti-Ferroptosis, Anti-Glycation, and Antioxidant Activities Protect Human Keratinocytes in A Cell Death Model with Erastin and Reactive Carbonyl Species

Huifang Li[1], Ni Deng[1,2], Tess Puopolo[1], Xian Jiang[3], Navindra P. Seeram[1], Chang Liu[2]*, Hang Ma[1,3]*

[1] Bioactive Botanical Research Laboratory, Biomedical and Pharmaceutical Sciences, College of Pharmacy, University of Rhode Island, Kingston, RI 02881, USA; hang_ma@uri.edu

[2] Proteomics Facility, College of Pharmacy, University of Rhode Island, Kingston, RI 02881, USA; hichang813@uri.edu

[3] Department of Dermatology, Laboratory of Dermatology, Clinical Institute of Inflammation and Immunology, Frontiers Science Center for Disease-related Molecular Network, West China Hospital, Sichuan University, Chengdu 610041, China; jiangxian@scu.edu.cn

* Chang Liu (hichang813@uri.edu); Phone: +1-(401)-874-2924
Hang Ma (hang_ma@uri.edu); Phone: +1-(401)-874-2711
Address: 7 Greenhouse Road, Kingston, RI 02881, USA

Figure S1.

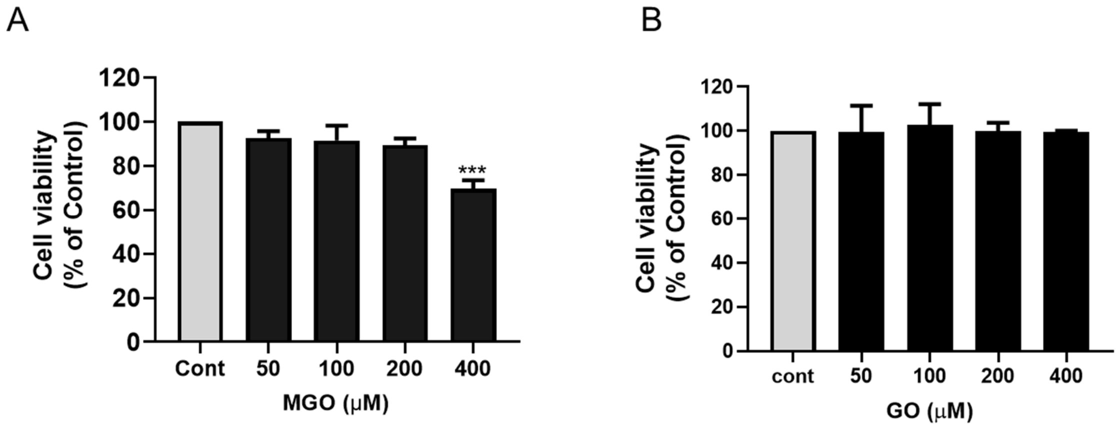

Figure S1. Effects of MGO and GO (50-400 μM) on the cell viability of HaCaT cells.

Figure S2.

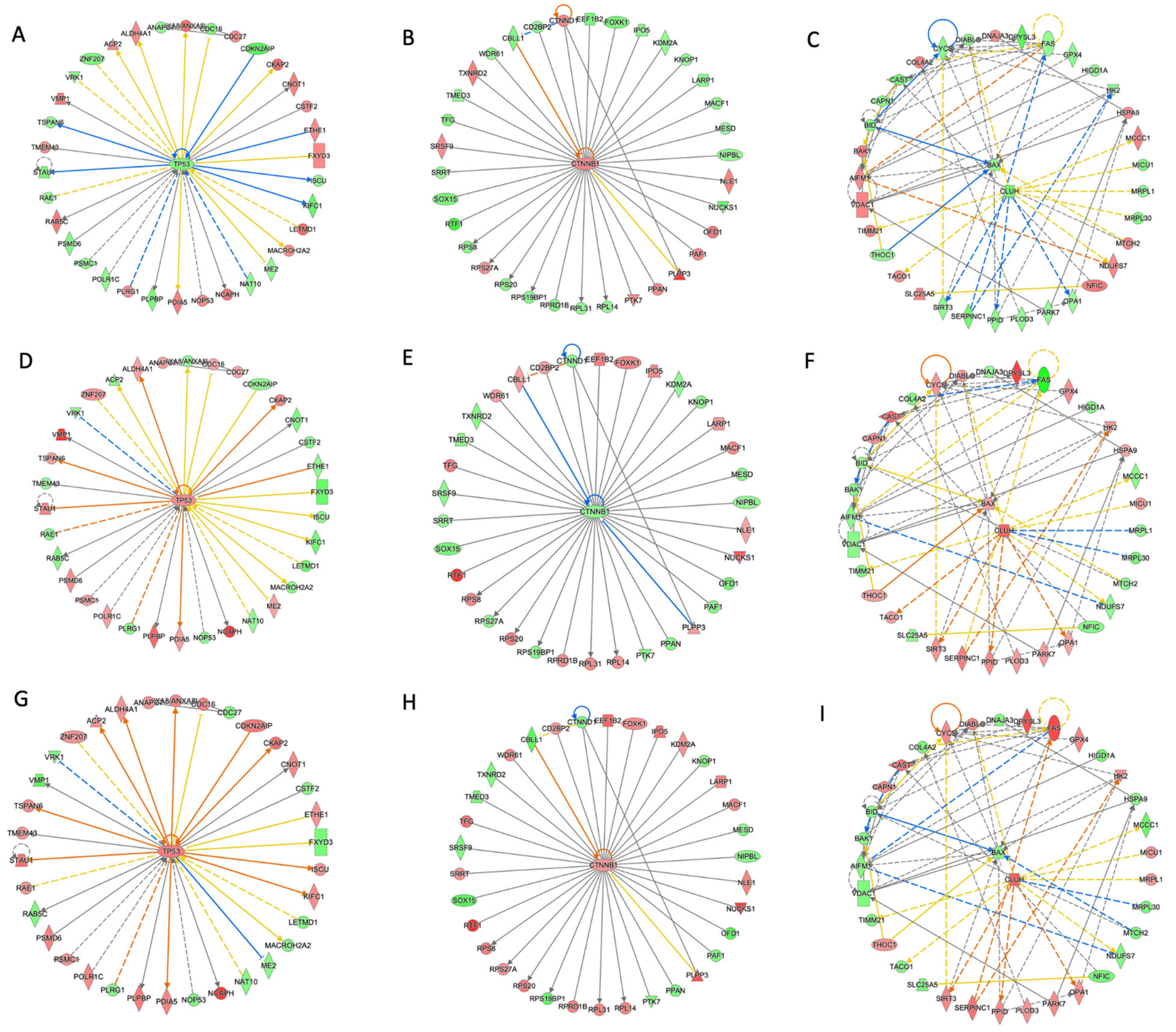

Figure S2. Networks related to cell death and survival. Three networks were selected from the 25 networks generated by IPA analysis of our proteomics dataset based on their relevance to cell death and survival. Each network consists of 35 molecules represented by nodes in the radial graph. Green nodes represent downregulated molecules, while red nodes represent upregulated molecules. Network A, D, and G revealed the molecules involved in the cell cycle, cell death and survival, and embryonic development in the Erastin-induction, Erastin-GO induction, and

Erastin-MGO induction groups, respectively. Central molecule TP53 was suppressed in the Erastin-induced cells, whereas it was activated in the presence of GO or MGO. Network B, E, and H revealed the molecules involved in cell death and survival, DNA replication, recombination, and repair in the Erastin-induction, Erastin-GO induction, and Erastin-MGO induction groups, respectively. Central molecule CTNB1 was activated in the Erastin-induced cells, whereas it was suppressed in the presence of GO or MGO. Network C, F, and I revealed the molecules involved in cell death and survival, embryonic development, and organismal injury and abnormalities, in the Erastin-induction, Erastin-GO induction, and Erastin-MGO induction groups, respectively. Central molecules CLUH and BAX were suppressed in the Erastin-induced cells, whereas CLUH and BAX were activated in the Erastin-GO induction group. In the Erastin-MGO induction group, CLUH was activated, and BAX was suppressed, suggesting different regulatory mechanisms between Erastin-GO and Erastin-MGO treatments.
